# Patient and provider perceptions of a comprehensive care program for HIV-positive adults over 50 years of age: The formation of the Golden Compass HIV and aging care program in San Francisco

**DOI:** 10.1371/journal.pone.0208486

**Published:** 2018-12-05

**Authors:** Meredith L. Greene, Judy Y. Tan, Sheri D. Weiser, Katerina Christopoulos, Mary Shiels, Allison O’Hollaren, Eva Mureithi, Loren Meissner, Diane Havlir, Monica Gandhi

**Affiliations:** 1 Division of Geriatrics, Department of Medicine, University of California San Francisco, San Francisco, California, United States of America; 2 Division of Prevention Science, Department of Medicine, University of California San Francisco, San Francisco, California, United States of America; 3 Division of HIV, Infectious Diseases, and Global Medicine, University of California San Francisco, San Francisco, California, United States of America; SEISIDA (Spanish AIDS Society), SPAIN

## Abstract

**Objective:**

People living with HIV (PLWH) are living longer and developing comorbidities and aging-related syndromes. New care models are needed to address the combined burden and complexity of HIV and its comorbidities in this group. The goal of this study is to describe qualitative data from patients and providers that informed the development of a comprehensive care model for older PLWH.

**Methods:**

Patient and provider perspectives on the clinical care and service needs of patients living and aging with HIV were explored via surveys and focus groups at a safety net HIV clinic in San Francisco. We surveyed 77 patients and 26 providers and conducted separate focus groups of older patients living with HIV (*n* = 31) and staff (*n* = 20). Transcripts were analyzed using thematic analysis. Themes for a care program were additionally explored using findings from the literature on HIV and aging.

**Findings:**

Themes from surveys and focus groups emphasized (a) the need for knowledge expertise in HIV and aging, (b) focus on medical conditions and determinants of health of particular import (e.g. marginal housing) among older PLWH, (c) co-locating specialty services (e.g. cardiology, geriatrics) with primary care, and (d) addressing social isolation. Findings informed the design of a comprehensive, multidisciplinary care model for PLWH called the *Golden Compass* program composed of four “points”: Heart and Mind (North), Bones and Strength (East), Network and Navigation (South), and Dental, Hearing, and Vision (West).

**Conclusion:**

Based on patient and clinic staff perspectives from surveys and focus groups, we designed a multidisciplinary program of integrated primary and specialty care, as well as housing and social support, to address the needs of older PLWH within a safety-net infrastructure. *Golden Compass* launched in 2017 for PLWH older than 50 years. Future research to evaluate the effectiveness of this care program in improving patient outcomes and satisfaction is ongoing.

## Introduction

The population of people living with HIV (PLWH) on antiretroviral therapy is aging and experiencing a widening spectrum of aging-related diseases and conditions. Estimates indicate that by 2030, over 70% of PLWH in the United States will be 50 years or older.[1, 2] PLWH are at increased risk for other co-morbid diseases at relatively earlier ages such as cardiovascular disease, osteopenia/osteoporosis and various cancers, [3–9] all of which are likely to require subspecialty care. In addition, many PLWH exhibit multimorbidity, polypharmacy, and geriatric syndromes like frailty and functional impairment at earlier ages than seen in the general population.[10–16] The combined burden of HIV, comorbidities and geriatric conditions in this burgeoning population necessitates a shift in the current HIV clinical care paradigm beyond just achieving virologic suppression to one geared towards thriving at older ages.

Currently, in San Francisco, 63% of PLWH are over the age of 50 years.[17] The Ward 86 clinic was one of two HIV clinics in San Francisco to take part in a demonstration project focused on performing geriatric assessments among older adults with HIV (also known as the “Silver Project”) from 2012–2014.[16] Participants underwent several different surveys and assessments, with results entered into the medical record for primary care providers to review. This study found that, among 359 PLWH over the age of 50, 40% reported a fall in the past year; 39% reported functional impairment (needing help with at least one Instrumental Activity of Daily Living (IADL) such as managing medications or shopping); and 34% had an abnormal cognitive screening test. In addition, 58% reported experiencing loneliness,[18] 50% felt that they had low levels of social support, and 55% had at least mild depressive symptoms.[16] These findings indicate the need for designated clinical care programs for older adults with HIV that can address both well-described medical co-morbidities and geriatric conditions such as frailty, social isolation, and cognitive problems.

To inform the development of a novel patient-centered clinical care program for patients over 50 living with HIV at Ward 86, we conducted surveys and focus groups with key stakeholders, including patients and clinic staff, on the most important health issues or needs facing older adults with HIV. We additionally asked about services or components that should be included in a program and elicited recommendations for the branding of such a program. We further refined our model of care by incorporating principles from geriatric medicine, which stresses optimizing physical function and social and psychological health with medical care.[19] Finally, we took into account findings from the literature on the most frequent comorbidities seen among older PLWH [3–9] to inform our program. Based on this data, we describe the development of a comprehensive clinical care program that addresses the increasing complexity of providing care for older PLWH.

## Materials and methods

### Study design and setting

Ward 86 is a Ryan White and public health-funded safety-net HIV clinic located on the campus of San Francisco General Hospital (SFGH) that provides HIV primary care and other services to approximately 2,600 PLWH, who are either publically insured or uninsured. The clinic offers multidisciplinary services including social work and substance use counseling, clinical pharmacy services, nutrition counseling, and other subspecialty services including psychiatry, dermatology, and pulmonology. To inform a designated HIV and aging care program, we conducted surveys and organized focus groups of HIV-positive patients receiving care at Ward 86 and clinic providers and staff. The surveys were designed to gain both patient and provider/staff perspectives on experiences with the prior demonstration project (Silver Project) to understand what worked well and what could be improved upon in a new program, and to identify how the clinic could further address the needs of older patients living with HIV. We conducted separate focus groups of patients and providers/staff in order to explore in depth the needs of older adults living with HIV. Results from the surveys were incorporated into the interview guides as prompts for focused questions on services and to ensure that barriers identified in surveys were further explored. The UCSF Committee on Human Research (IRB) approved all study procedures.

### Survey participants

We conducted anonymous surveys of patients, clinical staff, and providers. Eligible patient participants were those living with HIV who were age 50 or older, English-speaking, and receiving primary care at Ward 86. Patient participants consecutively enrolled via self-referral from flyers available in the clinic between March and April 2016. All staff (e.g., administrative, nursing, medical assistants) and providers (NPs, MDs) employed at Ward 86 at the time of the study were eligible for completing the surveys. We obtained verbal consent from all participants interested in participating in the surveys, and we offered a gift card for participation.

### Survey procedures

A research assistant administered the patient questionnaire in a private room at Ward 86. The patient questionnaire gathered basic demographic information such as race/ethnicity, gender, and risk factors for HIV acquisition. The questionnaire contained ad-hoc measures of overall satisfaction with the clinic (assessed on a Likert-type scale of very satisfied to very dissatisfied), knowledge of aging-related initiatives within the clinic (yes/no responses) along with a validated item of self-rated health assessed using a Likert-type response scale (excellent, very good, good, fair, poor).[20] An open-ended item, “to provide comments or suggestions on how the clinic can improve care for older adults” was also included. Patients who had participated in the prior demonstration project, Silver Project [16], at Ward 86 additionally responded to ad hoc measures about how they perceived the embedding of these geriatric assessments into standard care paradigms at Ward 86 for patients over 50 years of age. This included questions about satisfaction with the length of time to complete the geriatric assessments (assessed on Likert-type scale of very satisfied to very dissatisfied) and which assessments they felt were the most useful/important (Check all that apply to “which aspects of the Silver Project Screenings were most useful/important?”).

Providers and staff were emailed a hyperlink to an online questionnaire or were administered a paper questionnaire at a monthly provider meeting. The provider questionnaire included questions about profession (e.g., nursing, medicine, social work), length of time employed at the clinic, ad hoc measures about the familiarity with the work of the prior demonstration project [16], comfort level with providing care to older adults (responses of very comfortable to very uncomfortable), and the same open-ended item as on the patient survey asking to “provide comments on how the clinic could improve care for older adults living with HIV.” Primary care providers whose patients participated in the Silver Project were asked about their satisfaction with communication of the assessment results (very satisfied to very dissatisfied) and a yes/no question if the assessments informed the overall care of their patients. Primary care providers whose patients participated in the project were asked the same question as patients about which assessments were most important (Check all that apply, “which aspects of the Silver Project Screenings were most important/useful”).

### Focus group participants

Participants eligible to actively participate in focus groups included those at least 50 years of age and fluent in English. Patients were recruited by self-referral from flyers posted throughout the clinic with interested participants contacting the research team. Eligibility was confirmed over the phone, and patients were asked basic demographic information once potential eligibility was confirmed. Patient focus groups were organized by participant characteristics to enhance participant comfort and enhance sharing experiences.[21] This organization resulted in the following groups, reflective of the diverse clinic population: men who have sex with men (MSM); women, including transgender women; men who contracted HIV from injection drug use or heterosexual contact; and older age groups, ages 60s and 70s. The focus groups of older ages included a few PLWH who received care outside Ward 86 (to elicit best practices with other clinics). Participants represented a range of self-rated health and included patients with differing lengths of time living with HIV given the association between duration of infection and various health outcomes.[22]

We conducted six focus groups, four with PLWH and two with staff/providers. The two provider focus groups were conducted with a multidisciplinary team of clinic staff and providers. Providers and staff were recruited via email invitations. Providers were purposefully recruited to include different disciplines across medicine, nursing, and social work in each focus group. For both patient and provider groups, we tried to target around 10 participants each to incorporate different opinions and to allow adequate time for all participants to engage.[21]

### Focus group procedures

Semi-structured interview guides were developed using the planning model PRECEDE-PROCEED[23] as a general guide to structuring our questions to inform program design and incorporating responses to open-ended survey items to ensure those topics could also be explored. The PRECEDE (Predisposing, Reinforcing, and Enabling Constructs in Educational/Environmental Diagnosis and Evaluation) portion of the model outlines an approach to designing interventions assessing environmental, social, and behavioral factors that may influence outcomes and has served as a framework for health promotion and implementation science interventions.[23, 24] For example, this model informed our inclusion of questions pertaining to barriers and facilitators of care and considering how branding would influence participation in services. The interview guides focused on three overarching topic areas: (a) General perspectives on important issues facing older adults living with HIV, (b) barriers to and facilitators of the ideal patient experience for older PLWH, and (c) perspectives on naming or branding of programs focused on services for older adults with HIV. Interview guides were developed separately for the patient and provider groups.

Focus groups took place in a private conference room in the same building as the clinic with two of the authors (M. Greene, LM) serving as facilitators. Participants were welcomed and seated as they arrived, and verbal consent was obtained. Focus groups lasted between 60 and 90 minutes. Participants in patient focus groups received $20 for their participation. Staff focus groups occurred over the staff lunch hour, and participants received lunch for their participation. All focus groups were audio-recorded and transcribed by a professional service. Participant names were removed from transcripts to protect participant privacy.

Two co-authors (M. Greene, JYT) read all transcripts and independently coded the transcripts using thematic analysis.[25] First, open coding was conducted by reading transcripts, developing analytic memos, and developing and assigning codes. Coders met regularly to discuss and revise, remove, or add new codes until consensus was reached and all transcripts were coded. Next, coders searched for themes among codes and reviewed and refined themes. Coding was done using Microsoft Word. To develop a clinical care model based on the data, clinic leadership met to discuss the focus group themes in conjunction with considerations around clinic infrastructure, flow, staffing, and logistics in drafting a clinical care model. The draft model was further developed through an iterative process, such that versions of the model were presented at staff/provider meetings for feedback, whereupon the model was refined and finalized by clinic leadership (M. Gandhi, DH).

## Results

### Survey findings

A total of 77 participants completed the patient questionnaire (35 of whom participated in the prior demonstration project, Silver Project). Overall, the median age was 58 (range 50–77), 69% were male and 49% were African American. Regarding level of satisfaction with care received at the clinic, 50 (65%) reported being very satisfied, 24 (31%) reported being satisfied, and 2 (3%) reported being dissatisfied. Demographic characteristics and self-rated health of survey participants are shown in Table 1. Thirty-five (44%) respondents were aware of a prior demonstration project focused on HIV and aging, which corresponded with those who had actually participated in the project. In total, 47 (61%) participants wrote in at least one response to the open-ended item “Provide comments on how the clinic could improve care for older adults.” The majority of comments (n = 20, 43%) were affirmative in nature, such as “very nice care here” or “the care you provide is pretty good.” Ten (21%) responded with comments regarding clinic flow and space concerns, including wait times for appointments, need for more social work staff, and the physical environment. Three comments specifically pertained to how the physical environment and wait times were challenging for those with mobility impairment. Seven (15%) responded with comments on addressing transportation issues to come to clinic, seven (15%) reported needing more mental health and support services to address stigma and isolation, and another 15% responded with comments on provider knowledge of aging and HIV issues, with two comments regarding how to promote aging services availability within the clinic.

**Table 1:** Demographics of patients by surveys and focus groups.

		***n*, (%) or (IQR)**	
		Survey (*n* = 77)^a^	Focus groups (*n* = 31)
**Age (years)**		58 (55–62)	59 (55–62)
**Length of HIV infection (years)**		20 (10–29)	21 (17–27)
**Gender**	Women	19 (24.7)	6 (19.4)
	Men	53 (68.8)	24 (77.4)
	Transgender	5 (7.8)	1 (3.2)
**Race/Ethnicity**	Black or African American	38 (50.6)	15 (48.4
	Non-Hispanic White	26 (33.8)	13 (41.9)
	Latino	9 (11.7)	5 (16.1)
	Other	6 (7.8)	-
**Possible mode of HIV transmission^b^**	Men who have sex with men (MSM)	32 (41.6)	18 (58.1)
	Heterosexual	30 (39.0)	11 (35.5)
	Injection drug use	22 (28.6)	7 (22.6)
**Self-rated health**	Excellent or Very good	28 (36.4)	12 (38.7)
	Good	32 (41.6)	10 (32.3)
	Fair or Poor	17 (22.1)	8 (25.8)

IQR, interquartile range.

^a^Including 35 participants of the Silver Project demonstration project.

^b^Participants could select more than one risk factor for HIV.

A total of 26 providers or staff members who work in the clinic completed the provider/staff questionnaire (10 who had patients participate in Silver Project); characteristics are shown in Table 2. The majority of staff and providers were familiar with the prior demonstration project (n = 23, 88%). Regarding level of comfort in providing care or services for older adults at the clinic, five (19%) reported feeling “very comfortable,” 13 (50%) reported feeling “comfortable,” and eight (31%) reported feeling neutral. Out of the 26 respondents, 12 staff and providers (46%) provided responses to the open-ended item “provide comments on how the clinic could improve care for older adults living with HIV.” Staff and providers (n = 4, 33%) noted wanting educational opportunities to learn more about management of comorbidities and aging issues, with one respondent also noting a desire to educate patients. Staff and providers focused on specific services they would like to see in the clinic including medication management/polypharmacy (n = 2, 17%), having regular geriatric assessments, and services (n = 3, 35%), and one respondent reported the need for memory evaluations. Two respondents (17%) noted staffing shortage concerns.

**Table 2:** Profession/Discipline of staff and providers by survey and focus groups.

	**Survey (***n* **= 26)**^**a**^	**Focus groups (***n* **= 20)**
**Medicine**	13 (50.0)	5 (25.0)
**Nursing**	6 (23.1)	8 (40.0)
**Social Work, Administrative Support, and Pharmacy Staff**	5 (19.2)	7 (35.0)

^a^10 of 26 had patients who completed assessments in the demonstration project.

Of the 35 patients who had participated in the Silver Project, 11 (31%) strongly agreed that the time to complete the geriatric assessments was acceptable, 22 (63%) agreed and two (6%) felt neutral toward the amount of time required to complete for assessments. Among primary care providers who had patients participate in the Silver Project, four of the 10 (40%) providers were very satisfied with communication of results in the medical record, five (50%) were satisfied and one felt neutral (10%). All providers who had patients participate in the program felt the geriatric assessment results informed the care of their patient and affected treatment plans. Patients and providers who had participated in the Silver Project ranked each of the assessments that had been incorporated in the project. Overall, assessments of depression and falls were ranked the highest—as the most important among patients and as most useful among providers (Table 3). Other priorities with overlap between patients and providers included assessments of memory, medication adherence, and either loneliness or social support.

**Table 3:** Rankings of health assessments by patients and providers/staff^a^.

Most Important By Patients (*n* = 35)	*n*	%	Most Useful By Providers (*n* = 10)	*n*	%
Depression	22	68.8	Falls	9	90
Falls	17	53.1	Memory	8	80
HIV Medication Adherence	17	53.1	Depression	6	60
Social Support	17	53.1	Functional Status	6	60
Memory	16	50	Loneliness	5	50
Anxiety	13	40.6	HIV Medication Adherence	5	50
Functional Status	13	40.6	Social Support	5	50
Gait Speed	10	31.3	Gait Speed	4	40
Loneliness	9	28.1	Chair Stands	3	30
Chair Stands	9	28.1	Substance Use	3	30
Substance Use	7	21.9	Anxiety	2	20
PTSD^b^	6	18.8	PTSD^b^	1	10
Abuse	6	18.8	Abuse	0	0

^a^Only respondents who participated in the past demonstration project answered this question so overall n is smaller than for overall survey sample.

^b^PTSD, post-traumatic stress disorder (symptoms of post-traumatic stress)

### Focus groups findings and themes that emerged

A total of 31 patients participated in the four patient focus groups, and a total of 20 staff and providers participated in the two staff focus groups. Tables 1 and 2 provide the overall demographics of focus group participants. The four patient groups included the following number of participants: 10 in the MSM group, seven women, eight heterosexual men and IDU, and six in the final group. The two provider groups consisted of 12 and eight participants each with a mix of nursing, medicine, social work and other disciplines. Only two patients who initially expressed interest declined to participate, both of whom were white MSM, due to lack of time/change in interest. Overall, four themes relevant to a new HIV and aging care paradigm at Ward 86 emerged from the focus group discussions with both patients and providers: 1) Knowledge of HIV and Aging Topics; 2) Specific Needs for Older Adults Living with HIV; 3) Importance of Social Networks; and 4) Need for Integrated HIV and Specialty Aging Services. A cross-cutting theme was navigating the changing and increasingly complex care needs of older adults living with HIV.

#### Provider and patient knowledge of HIV and aging topics

A prominent theme from both patient and provider focus groups was the appreciation that providers needed to have a deeper knowledge base to care for older adults with HIV. Patients expressed a desire to understand more about HIV and aging issues generally. As one patient indicated, providers should assume that they know very little about getting older with HIV:

*“I think [our healthcare providers] should presume that we know less rather than more*, *as we get older we've had experiences of what comes with the condition, what comes with age, what comes with a combination of the two and it's better to presume less rather than more knowledge.”*

Patients also expressed a desire for education on management of other chronic conditions and ways to improve overall self-management for chronic conditions other than HIV, as one patient explained:

*“I think it would be a good thing if we were taught how to check our sugar, check our pulse. I mean, the pharmacy gave me a box [glucometer] and all this equipment that goes with it*. *But I didn't know how to use it. I'm just sitting there looking [at it]. It looks like a phone to me, right? So in order to find out how to use that, I had to first make an appointment and then second, wait until my appointment was ready.”*

There was uncertainty among patients who felt they were not always sure whether their health issues were from HIV, from aging, or from long-term use of antiretroviral therapy (ART), as one patient expressed:

*“I have lived with HIV for an extremely long time*, *[I don’t know about] the long-term effects and consequences of that later in life, as well as being on antiretroviral medicines.”*

Patients expressed concerns regarding the long-term effects of living with HIV as well as long-term effects of being on antiretroviral therapy. One participant referred to concerns regarding the distinction in the long-term effects of living with HIV vs. long-term ART use:

*“There's two streams, there's the having lived with HIV/AIDS for an extremely long time, there's effects and consequences of that later in life, as well as being on antiretroviral, those two combined*, *those would be problematic for a clinician.”*

Nevertheless, patients also expressed a desire to ensure providers knew how to manage both HIV and aging-related health concerns, particularly in acknowledging the potential effects of ART on health:

*“Our physicians need to be educated on the effects of the medicines. Speaking to my physician about the fatigue, the arthritis and stiffness and the weight gain, she brings it back to it being at my age, a lot of these things are wear and tear. It's not so much the HIV. But a lot of it is relative. There's a big difference of aging between a regular chronologically aged person and a person living with HIV. So I think the education would be a service. Or where the doctors know what*, *you know, rather than me telling the doctor that I think that this is due to my HIV med, and them denying that.”*

Patients expressed concern regarding the lack of an integrated and comprehensive approach to their health as they age and the missed opportunities for diagnosis and treatment:

*“But there was another doctor…he was an excellent HIV [provider], but as I aged, he overlooked the fact that I might have heart disease in my history*, *and that I might be a diabetic. So he wasn't really testing me for those diseases as I was aging, because his concern was to keep me undetectable. So, you know, these two doctors had specialties that didn't really combine and they didn't interact very much. So I was kind of at a loss, and that's why I think there needs to be a combination of physicians who are trained for both… and are looking at disease as we age, not just because we have HIV or AIDS, but there needs to be some kind of a focus on the whole picture of the patient as they age. And there are also predispositions with HIV patients who get these diseases faster than later, because of our meds and the HIV, it makes us more susceptible to getting disease earlier and chronic disease.”*

Providers expressed a need for more training to increase their knowledge on treating patients who are aging with HIV. Suggestions for methods by which to increase provider knowledge base included a geriatrics “boot camp” with workshop-based training. One provider explained:

*“One thing that particularly stumps me is being able to discern the additive effect of all these comorbidities on a single patient. They have vascular disease and [chronic obstructive pulmonary disease*, *or COPD] and probably a little dementia. I feel like I definitely need the boot camp but also I'm really appreciating that [geriatricians are] here because my sense is—one of the things I think geriatricians do well is [helping us understand] the cumulative effects of all these comorbidities.”*

#### Healthcare needs of older adults living with HIV

A number of overlapping health issues and needs were raised in both patient and provider/staff focus groups. Patient health issues and needs included neurocognitive screening, addressing falls and frailty, care navigation and case management, the profound impact of mental illness and marginal housing on those who are aging, and access to ancillary services such as dental, vision, and hearing.

A health concern in both patient and provider/staff groups was addressing cognitive changes. One patient described a need for integrating screening for cognitive changes as part of regular care:

*“Mental functionality as we get older is going to be an issue for some of us, and I think screening or however you check for early stage dementia or Alzheimer's is something that would be important [to incorporate into this program for us over 50]*. *Because you don't want to get to the point where you can't make those decisions and then realize you didn't.”*

A provider shared concerns regarding a patient who was experiencing cognitive decline that interfered with her care:

*“[O]ne of my oldest women who's 74, she's been having a lot of dementia and cognitive issues, and part of what had happened for her is she's been getting really confused and paranoid and fairly hostile and reactive when she has come in. And I think it's really interfered with her care and her understanding of what is happening. And it's disturbing, because she's quite a frail, elderly woman at this point, and has multiple other HIV-related and non-HIV-related health issues*.*”*

Frailty and falling were also common health issues for PLWH over the age of 50, and both groups discussed a need for programming to focus on raising awareness in preventing falls. One patient described frustration at not being informed that his falls were likely due to health issues such as high blood pressure:

*“A couple of years ago, I kept on falling and I was on the bus. I would just fall onto the floor. I didn't know what was going on. My doctor told me, ‘Oh, well you know*, *your high blood pressure is too high, you got to lower it down.’ And I didn't know, I told him, ‘You mean to tell me I was falling and I didn't know my blood pressure was causing me to fall?’”*

One patient suggested instituting exercise activities into a program for older PLWH as part of preventing falls and addressing other health conditions:

*“[I]t will be nice to have a place on Ward 86 to do physical therapy for people 50 and over just because I don't know, some of the medication, your bones get, I don't know*, *brittle. And also a place where you can do some exercise, light exercise and yoga, it really helps, I'm 51 but I experience a lot of stress sometimes.”*

There was high agreement and emphasis among patient and provider focus groups about the need for more case management and social services, especially for those who are marginally housed. Older patients living with HIV often have many medical appointments to keep. One patient explained:

*“[W]e have to go to so many different places, this is just the list of the doctors that I have. I mean it's at least 20, 25 of them. It's ridiculous and it's a lot to deal with. Yeah*, *because you can miss an appointment and then you're screwed, you're set off until months later to get to [see] some of these [doctors]. Referrals are good. And the lady said I didn't need a referral at this particular place on Geary Street… But she said, eventually you'll need a referral so I've got to work on that to get that ready. And it's all a process. It would help [if we could have] at least somebody in between [who can] guide you where you need to be going.”*

Patients pointed out that the inability to access timely case management becomes problematic for accessing services like housing and food:

*“[T]hey could use some more social workers because they're always jam packed in there and busy*. *A lot of times, say, you get a letter from the food bank and they want you to come in with labs and a letter of diagnosis and all that stuff every six months—but you don't see your doctor for another month.”*

Better access to ancillary services was a resounding theme among patient focus groups. Many patients expressed frustration that centered on being able to access dental and vision care:

*“What I don't understand about healthcare [is that] your dental is just as important because it has a lot to do with your overall health. With your dental, I got to go through all these hoops and hurdles*. *Teeth have a lot to do with your health but…I have to write a [letter] to [organization offering free or low-cost dental care] for acceptance as to why I should be in the program.”*

#### Building social networks to address social isolation and loneliness

Another emergent theme was social isolation and loneliness among older PLWH and a need for regularly held social gatherings and events. A clear consensus emerged among patient and provider focus groups regarding patients’ need for improved social support networks. One patient suggested:

*“We should have…somewhere we can go and socialize…have lunch and have social workers there, if we need to get stuff done. For me, I live alone*, *I have friends but sometimes my friends work and I like to get out and be around other people socially during the day, I have groups but they are in the evening, but in the daytime I'm just stuck at home. We need somewhere we can just go and, you know, have lunch or somewhere to socialize at.”*

Patients had several suggestions for the types of activities that a program for older PLWH could host. These activities included exercise classes (e.g., yoga, tai chi). One patient explained that living on limited income often prohibited him from socializing and that outdoor social activities organized by the program would help to address that:

*“I'd say organized outdoor walks*, *stuff that promotes [being] outdoors, organized through the program would be a great way to keep people from being sedentary. At 900 bucks a month, after I pay rent, my utilities and everything, I'm not left with a lot of money to go out.”*

Patients described a desire for organized opportunities to interact with other older adults living with HIV as an important way to help address isolation. Many older PLWH feel isolated due to the stigma of being HIV-positive:

*“When I first was told that, that I was HIV, I wanted to kill myself … But now as time went by, I'm happy that I'm alive and…I feel in my heart that this group and people getting together*, *oh god, it's strength, it's so much strength, I mean without it, I don't know what I would do. I'll be in my room a lot of times and I'll just be thinking a lot, I do a lot of thinking [alone, but] it's best to let it out instead of holding it in because when you hold stuff in, it kills you more and when you let it out, you feel free, because you got the load off your shoulders.”*

In general, patients felt having peer support groups would provide enormous benefits and a welcome opportunity to share resources. Discussions in patient groups reflected patients’ eagerness to share knowledge and gather tips among peers. One patient commented about the helpfulness of attending the focus group for gathering knowledge regarding services:

*“All the things that we've discussed here …I didn't know about the orthopedics place*, *some people didn't know about the eye thing.”*

Similar themes were raised among provider/staff groups regarding social isolation and loneliness among older PLWH. Providers discussed how patients who have outlived the HIV epidemic are left isolated and in need of social connection:

*“Well, certainly, as I pointed out, the sense of isolation is heightened for HIV seniors because the epidemic has decimated their friends and connections*. *That's not the case with just the general population that would be HIV negative. Their friends were not impacted in such a dramatic way.”*

One provider explained women often experience a profound sense of isolation due to the loss of kinship in the context of HIV stigma:

*“In terms of the communities they've lost, I think for women with HIV that I work with, the stigma is still so profound for them, that's what really feeds a lot of their isolation*. *And a lot of them want to be very family and community-based, but they feel stigmatized about the HIV. Otherwise, health-wise, I think they share with the non-HIV population, you know, the same kinds of issues.”*

Providers specifically identified issues they observed among “long-term survivors of HIV.” The consequences of long-term survivorship include the lack of financial security and long-term stability. Providers expressed concerns for patients who are often long-term survivors left with very little in terms of finances and other resources:

*“People that age with HIV usually have been HIV positive for many years, so they've gone through the initial earlier parts of the epidemic in their twenties or thirties*. *And I just realize how much of your resources later on in life really has to be developed or built at those ages, because if they dropped out of school or didn't build up those, you know, credentials and abilities, then when they get to the sixties and seventies, they may not have family. They don't have, you know, savings. They don't have a retirement plan. And, you know, so we're—it's very difficult. It just struck me how little reserve they have at that age, and that just seems to put them at a much more disadvantage than any other patients that I've had to take care of. So it's very sad.”*

#### Need for “blended,” integrated primary, geriatrics and specialty care

Themes derived from both patient and provider focus groups underscored the perceived need for a comprehensive HIV-aging clinical care program that “blends” primary care with both geriatric consultation and specialty care for comorbidities most relevant to older HIV-infected patients (e.g., accessing cardiologists on-site). One patient noted:

“*[The program should] incorporate all these things*: *pharmacy care*, *social care*, *primary care physician and all of it working seamlessly*, *it would be less work for the clinic…if it's staffed right*, *having something for 50 and older…would really work well*.*”*

Patients and providers both appreciated the benefits of co-locating additional care paradigms for older PLWH right in the clinic instead of making outside referrals for additional care components. As one patient explained:

*“Referrals outside of the practice for a specialty are always problematic as we all know and it's just difficult to manage, if somebody is doing that for me*, *which I don't like, you're never really sure what you're going to end up—if you get into a specialty that may not be fully aware of HIV and certainly not HIV and aging…The referral not to just say cardiology….cardiology at UCSF but a referral to cardiology would be not just a referral to the cardiology department but somebody who knows HIV cardiology and I mean even more specialized, you know, aging.”*

A provider sympathized with the difficulty her patients experience having to travel to and from various appointments that are located across distances:

*“I think it's hard enough for young patients to go over to the hospital for various appointments. So I mean, just being able to centralize the clinics somehow. Maybe even, you know, Lab, Radiology*. *I mean, I don't know if they have enough room in the main—the whole hospital building. But it seems like it would be nice if we could just shorten the <laughs> distances they have to travel.”*

Providers expressed the need for a clinical care model that adequately addresses the complexity of providing care to older PLWH. Providers care for patients with intersecting social and clinical health needs that extend beyond the standard of care and treatment they can provide. One provider explained:

*“[Many of] my patients are elderly, but cancer itself makes life complicated, and they're HIV-positive on top of that. This patient of mine*, *he's also over 70 and was diagnosed with early stage anal cancer. Medically it's a pretty straightforward thing. You know, you get 5 to 6 weeks of chemo and radiation, and at his stage, the cure rate is probably in the 90 percent range. Medically it looks very simple, but then when you go into the logistics of treating this person, his housing is very unstable. He had been, you know, on the streets many times over years. Part of the reason could be drug use and maybe the HIV as well.”*

## Discussion

As the population of PLWH grows, providers face challenges in caring for aging PLWH that include managing multiple comorbidities and aging-related syndromes.[19] To inform a comprehensive care program for PLWH at a large safety-net urban HIV clinic, we conducted surveys and focus groups of both patients and providers to determine the most important components of a comprehensive HIV and aging care program for PLHIV over 50 years. We found that a diverse group of patients and providers agreed on many priorities for the aging HIV population, including assessments of falls, depression, and memory concerns. These same findings also emerged in the focus groups. Patients and providers both felt knowledge of HIV and aging issues was important, as well as components to address the unique social challenges faced by older patients with HIV. Patients and providers both discussed a need to have integrated HIV and aging services, a concept that has recently emerged in the HIV literature and dubbed “Geriatric-HIV Medicine.”[19, 26] Overall, our findings support and add to prior qualitative work on patient perspectives on the needs of older adults living with HIV and provide new data on provider perspectives, which is limited in the literature.

Survey and focus group results complemented each other. Survey results highlighted important similarities between patients and providers on the perceived needs of older adults living with HIV. Patients and providers agreed that assessment of both depression and falls was important, as was testing for neurocognitive deficits (memory decline) and social isolation (loneliness, social support). In response to a request for comments on how to improve care, providers focused more on medical services and education needs, while patients noted barriers such as wait times and transportation as areas for improvement. Focus groups expanded upon survey results. Cognition, falls, and isolation all emerged within the focus groups to be important areas to address. Of note, transportation was important among survey respondents but had less emphasis among focus group participants, although patients in some groups did mention it. Issues such as housing security emerged more in focus groups, an issue which has been well described as affecting HIV outcomes such as engagement in care and viral suppression.[27] As Ward 86 serves as a safety net for publicly insured population, unstable housing is a significant concern (occurring in 38% of our overall clinic population), so the discussion around this issue in focus groups was not surprising.

We found interesting results regarding the importance of patient and provider knowledge of HIV and aging issues, especially among providers. That is, while surveys showed that patient satisfaction was high and providers were comfortable in treating older patients, themes from both patient and provider focus groups nevertheless indicated that providers needed more training and a stronger knowledge base on aging and HIV. Additionally, among the four providers who commented that additional education was needed in the surveys to improve care, all rated their comfort level with providing care to older adults as “comfortable” or “very comfortable”. While one explanation may be that providers will not readily admit they feel uncomfortable, these findings may not have to be viewed as mutually exclusive. In healthcare, especially in a university-affiliated setting, the culture is often of continuous learning and skill improvement. Although providers may feel comfortable, they may also desire additional training.

The themes of knowledge and importance of social networks that emerged in the focus groups and reported in surveys is consistent with prior qualitative research among older adults living with HIV. Both a prior study of older adults living with HIV in Ontario and a 2010 needs assessment of older adults with HIV in the San Francisco Bay Area identified healthcare providers’ knowledge of aging issues as a concern.[28, 29] The Ontario study also described patient uncertainty around whether health issues stemmed from HIV, aging, or antiretroviral therapy, a theme that emerged in our groups as well.[28] We are unaware of prior studies on HIV providers’ perspectives on aging/geriatrics knowledge.

In general, focus group themes around specific healthcare needs for older adults living with HIV and importance of social networks reflect many of the challenges that have emerged in the HIV and aging literature. Focus groups emphasized management of comorbid (e.g. diabetes, cardiovascular disease) and geriatric conditions (falls, frailty) that are common among older adults living with HIV.[10, 13] Addressing sensory impairment through improved access to ancillary services like vision and dental care was also reported. Social isolation and loneliness among older adults living with HIV are well described in the literature, [10, 18] and our findings support that these are critical issues to address. Patients discussed possible solutions that could both promote healthy lifestyle and decrease isolation such as exercise classes in addition to support groups and activities to promote a sense of community. The need to engage in meaningful activities and find ways to foster social supports is similar to findings in prior studies.[29,30] Importantly, our study also demonstrated that providers want to find ways to address the challenges of isolation they see among older patients. It is important to note that the themes of specific services as well as loneliness and isolation emerged across all groups, including women, MSM, and others. Reasons why older women and MSM feel lonely may differ as alluded to in quotes from providers, and a future study will provide an in-depth exploration of how different groups of older adults living with HIV view these service needs and issues.

Both patient and provider focus groups provided rich, contextualized findings regarding the need for an integrative approach to geriatrics and specialty care within the HIV care setting and as such, helped address questions posed in a recent editorial on Geriatric HIV Medicine.[26] Geriatric assessments such as assessment of physical function and frailty have now been shown to predict outcomes among PLWH, [31,32] and our findings support that patients and providers would welcome these assessments in clinic as well as strategies to address geriatric conditions such as falls and cognitive issues. Regarding how to integrate services, patients in particular, but also providers acknowledged the importance of co-locating services especially for those with limited mobility. Our findings support a prior study of Ryan White HIV/AIDS Program-funded clinics, which noted the importance of co-location of services, provision of comprehensive services including mental health care, and a clinic culture that reduces HIV stigma.[33] The emphasis was not specifically on care models for older adults, but discussed the need for comprehensive, interdisciplinary care from the perspective of clinic administrators and providers.[33] Our findings add to this by showing that both patients and providers support these concepts. Our findings also suggest how Ryan White HIV/AIDS Program-funded clinics such as Ward 86 can ideally care for older adults living with HIV, especially with an emphasis on wrap-around services and team care, concepts critical to both HIV and geriatric medicine.

The major limitation in our design was that the focus group and survey data came from patients and providers from a single clinical site. Moreover, our focus groups excluded non-English speaking patients so the results may not be generalizable to monolingual Spanish speakers. Although groups did include bilingual and Latino participants, close to 8% of the overall clinic population may be monolingual Spanish speakers. We plan to address this gap by conducting focus groups with native Spanish speakers in the future. Finally, although we did include participants living with HIV for different lengths of time, the majority of the patients recruited for the focus groups were long-term survivors (median length of HIV infection 20 years), who may have different needs than those who are newly diagnosed after age 50.

### Golden compass, a novel clinical care program

The overarching purpose of the surveys and focus groups was to inform a new program at Ward 86 on HIV and aging. Combining this data with a literature review on the most important comorbidities and psychosocial issues facing older PLWH, [3–16] we designed a novel clinical care program for PLWH over the age of 50 years called the “Golden Compass” at the Ward 86 Clinic in San Francisco. The structure of this clinical care program stems from unifying principles in both HIV medicine and geriatrics care to comprehensively assess the complex care needs of an individual, to address psychosocial needs concomitantly with medical needs, to truly provide “patient-centered” care, and to co-locate and facilitate services to reduce the burden on the individual in maintaining and improving his/her health. Of note, the name of the program was derived from the patient focus groups where “Golden Years” was an acceptable term referencing aging and the need for navigation of the healthcare system an overarching theme. The name was iteratively assessed in patient, staff, and provider groups thereafter with positive reviews.

**Fig 1:**
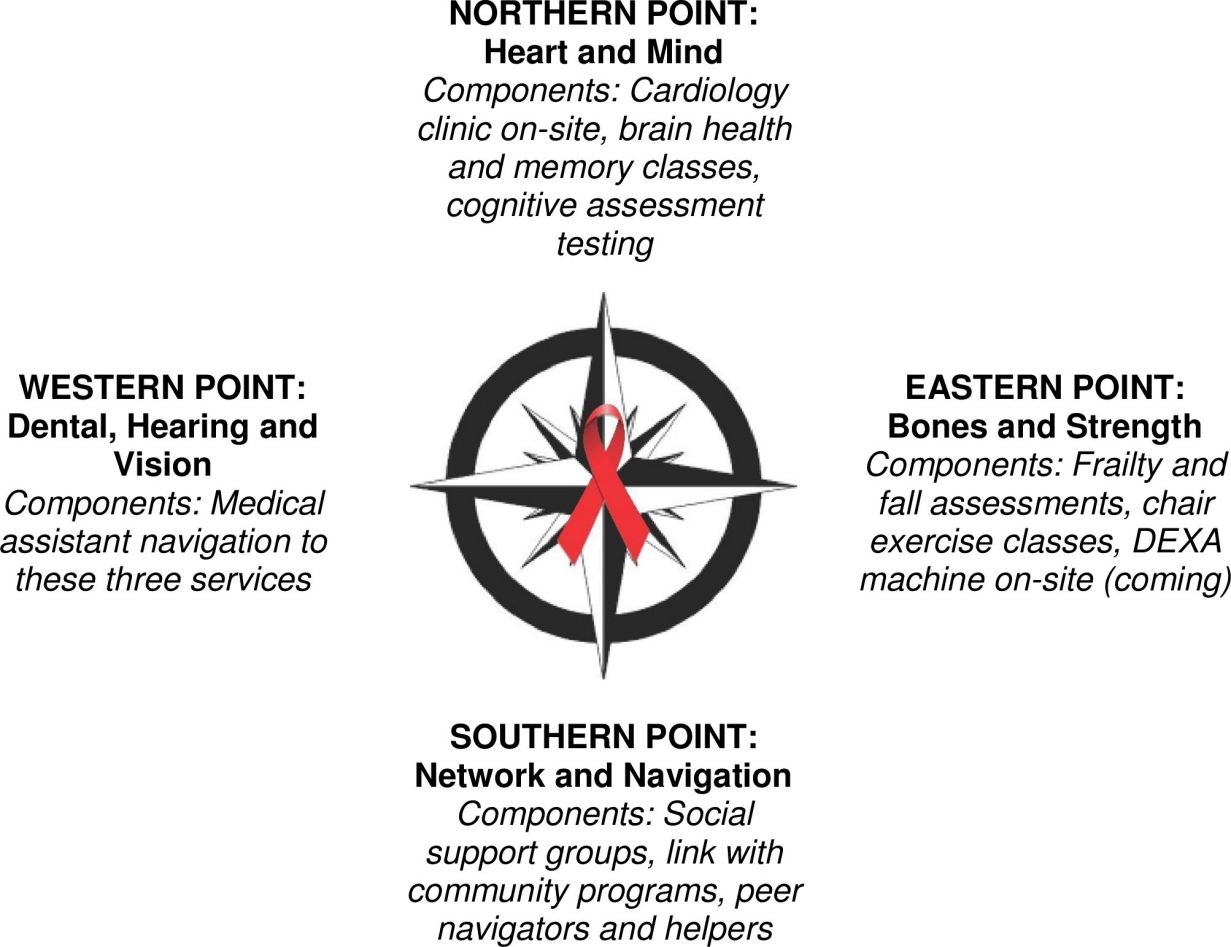
Overview of the Ward 86 golden compass program.

Based on the themes from the focus groups, findings from the surveys such as the most important assessments ranked by patients and providers, as well as practical considerations of local resources, we designed the “Golden Compass” program at Ward 86 to focus on four pillars of care or “points” of a compass (Fig 1): 1) **Heart and Mind (Northern Point)** provides comprehensive cardiac, psychiatric, and neurocognitive care, including on-site cardiology, cognitive evaluations, and memory classes; 2) **Bones and Strength (Eastern Point)** focuses on bone health, strength, fitness, and addressing frailty and functional decline, including on-site geriatrician consultation once weekly at Ward 86; 3) **Dental, Hearing and Vision (Western Point)** ensures clients have appropriate screenings and are linked to dental, hearing, and vision services to address sensory impairment; 4) **Networking and Navigation (Southern Point)** coordinates with community partners, as well as provides peer support groups at Ward 86, along with other social and community-building activities. The Southern point also focuses on the provision of emergency housing to program participants over age 50 with marginal housing, given that unstable housing is likely to have a heightened impact in older individuals who need help with permanent housing, and a provision of legal services for our clients through a local partner agency. Additionally, given the importance of provider and staff knowledge that was found in surveys and focus groups, staff have attended local Department of Aging trainings. Providers will gain knowledge through recommendations made by the on-site geriatrician, and further efforts are underway to develop formal trainings for staff and providers. The Golden Compass program launched in January 2017 and program tracking, evaluation, and monitoring is ongoing. If the program is replicated at other sites, local refinements to meet the needs of the aging population in that HIV clinic should be considered.

## Conclusions

In summary, we describe the formation of a comprehensive care program for older adults living with HIV based on survey and focus group data, geriatric assessments with our clinic population, and findings from the literature on HIV and aging. Our findings offer insights on integrating comprehensive geriatric assessment to inform geriatric HIV care programs, a critical question that needs to be addressed.[26] Program evaluation and monitoring is ongoing to assess whether patient outcomes and patient satisfaction with care have improved after the launch. Given the growing population of people living with HIV over the age of 50, the increasing complexity of comorbidities and psychosocial conditions in this group, and the accumulation of new data on how to effectively manage these conditions, HIV care programs should provide intensified care for their older patients to help them not just survive with HIV, but thrive.
